# A randomized clinical study to evaluate the effect of denture adhesive application technique on food particle accumulation under dentures

**DOI:** 10.1002/cre2.168

**Published:** 2019-06-17

**Authors:** Mounir Atassi, Kimberly R. Milleman, Gary R. Burnett, Susmita Sanyal, Jeffery L. Milleman

**Affiliations:** ^1^ GSK Consumer Healthcare Weybridge, Surrey UK; ^2^ Salus Research Inc. Fort Wayne Indiana USA; ^3^ Syneos Health Hyderabad India

**Keywords:** denture adhesive, food‐occlusion model, retention, stability

## Abstract

Food ingress under dentures is a common problem that may be reduced by denture adhesive use. The objective of this study was to explore the effect of the mode of application of a denture adhesive on reducing accumulation of food particles under dentures. This was a single‐centre, controlled, single‐blind, randomized, three‐treatment, three‐period, crossover study in participants with complete, removable well‐fitting, well‐made upper/lower dentures. Treatments were: 1) experimental denture adhesive application (test adhesive) applied with a precision applicator as continuous strips; 2) marketed denture adhesive (positive control) applied using a flat ribbon nozzle as dabs; 3) no adhesive. Food‐occlusion testing was performed by assessing peanut particle migration under dentures with denture retention/stability evaluated using the Kapur Index (Olshan modification). Differences were assessed using an ANOVA model. Adhesive oozing and perceptions of the adhesives were assessed by questionnaire. All 83 randomized participants completed the study. There were no significant differences between positive control or test adhesives versus no adhesive, or between test adhesive and positive control, for mass of peanut particles recovered from dentures. Both adhesives had significantly higher retention and stability scores compared with no adhesive (all *P* < .01). Participants reported significantly higher scores for denture comfort, confidence, satisfaction and movement with both adhesives versus no adhesive (all *P* < .01). No differences in adhesive ooze were reported between adhesives. No adverse events were reported. In conclusion, there was no difference in performance, as measured by peanut particle mass recovered from upper/lower dentures, for the test adhesive, positive control and no adhesive.

## 1 INTRODUCTION

Commercially available denture adhesives can enhance removable prosthesis treatment outcome by increasing retention and stability (Grasso, 2004). Clinical indications for the use of denture adhesives in conjunction with well‐made dentures include instances when anatomic structure is compromised or when neuromuscular control is impaired and affects a person's ability to develop the necessary adaptive muscle control for denture wearing (Zarb & Fenton, 2013).

In contrast to early denture adhesives, which were made from highly water‐soluble natural plant gums, modern denture adhesives comprise natural or synthetic polymers in combination with plasticisers and antimicrobial, binding and flavoring agents (Kumar et al., 2015). When hydrated with moisture or saliva, denture adhesives swell, filling the spaces between the denture and oral mucosa and providing strong cohesive bonds between the two (Kumar et al., 2015). This can result in improved denture retention and stability, which in turn improves chewing efficacy and ability, increases comfort and wearer satisfaction, and minimizes the accumulation and ingress of food particles between the denture and oral mucosa (Goncalves, Viu, Goncalves, & Garcia, 2014; Grasso, 2004; Kapur, 1967; Kumar et al., 2015; Munoz et al., 2012; Papadiochou, Emmanouil, & Papadiochos, 2015; Tarbet, Boone, & Schmidt, 1980).

Several studies have cited food entrapment or accumulation under dentures as one of the most common complaints in denture wearers, leading to pain and discomfort (Aghdaee, Rostamkhani, & Ahmadi, 2007; Brunello & Mandikos, 1998; Gosavi, Ghanchi, Malik, & Sanyal, 2013). In a large population‐based consumer research survey among Canadian denture wearers, the most common experience reported by the 2986 respondents was the perceived presence of food particles under the denture during mastication. Of the respondents, 25% reported regular incidents of food entrapment during chewing and 90% reported this occasionally (Data on File, 2017).

The ability of denture adhesives to restrict food accumulation under a denture has previously been explored in partial‐ and full‐denture wearers. Tarbet and colleagues used a qualitative subjective questionnaire and reported that participants perceived fewer food particles under their dentures with adhesive use (Tarbet et al., 1980). A number of studies have quantitatively measured the mass of food (peanut particles) that migrates under partial (Munoz‐Viveros, Schober, et al., 2011) and full dentures (Ahmad, Ibrahim, Hazmi, Tarib, & Kamarudin, 1990; Ahmad, Ibrahim, Hazmi, Tarib, & Kamarudin, 2010; Munoz‐Viveros, Tyson‐Johnson, et al., 2011; Munoz et al., 2012) during a chewing challenge. All of these quantitative studies, with one exception, (Munoz et al., 2012) reported statistically significant reductions in food particle entrapment under the denture when an adhesive was used. These studies also reported increased user comfort, confidence and satisfaction with dentures when using a denture adhesive compared with no adhesive. However, the ability of this methodology to differentiate between the effectiveness of different adhesive formulations or different techniques for adhesive application has yet to be demonstrated.

Currently marketed adhesives are provided as a dry powder, pre‐formed strip or denture cream adhesive. The cream adhesives are applied by extrusion through a nozzle onto the fitting surface of the denture. The pattern of application can vary between different brands of adhesives but typically falls into two classifications: spotting/dabbing the adhesive onto the denture or extruding a continuous/near‐continuous strip around the denture borders. In both cases, the adhesive is spread further between the fitting surface of the denture and the oral mucosa once the user has fit the denture. Logically, it might be expected that with the continuous strip method the adhesive is more widely distributed on the denture fit surface and located more tactically along the borders of the denture, leading to enhanced efficacy compared with the dabbing method.

Use of denture adhesive is not high among denture wearers. In surveys, of Greek, Dutch and Australian people, only around a quarter to a third reported ever using denture adhesive (Coates, 2000; Polyzois & de Baat, 2012). However, only around 5–7% currently used it. The main reasons for not persisting with denture adhesive use were that denture retention, chewing ability and comfort were not perceived to be significantly improved. Other reasons included that it was messy to use, that they did not like the taste and that it caused gagging or nausea. As such, refining both the taste and feel, along with application, is a vital part of denture adhesive development.

The aim of the current study was to explore if the pattern used to apply denture adhesive reduces the accumulation of food particles under dentures. Given that the methodology used here has been previously demonstrated to differentiate between treatment with an adhesive and no adhesive use (as detailed above), the primary objective was selected to ensure that the clinical model performed as expected. As such, this was to assess the efficacy of a standard marketed denture adhesive applied in evenly spaced dabs (positive control group) in reducing the ingress of food (peanut particles) under a denture compared with that of no adhesive (negative control group). Secondary objectives included assessment of the efficacy of an experimental denture adhesive applied using a continuous strip of denture adhesive (test adhesive group) compared with that of the negative and positive controls in the same clinical model for food occlusion, and assessment of the retention and stability performance of dentures while using the adhesives or no adhesive.

Exploratory objectives included assessment of participant preference for the two adhesives or no adhesive based on measures of denture stability, comfort, confidence and satisfaction with dentures while chewing peanuts, and assessment of participant preference for the two adhesives based on the measurement of adhesive ooze.

## 2 MATERIALS AND METHODS

This was a single‐centre, controlled, single‐blind (to safety and efficacy assessors), randomized, three‐treatment, three‐period, crossover study conducted in healthy participants with full upper and lower dentures (ClinicalTrials.gov identifier: NCT02928380). The study was conducted at a USA‐based clinical research facility. The study protocol was approved by an institutional review board (US IRB, Miami, FL, USA: U.S.IRB2016SRI/04) and was conducted in accordance with the Declaration of Helsinki and good clinical practice. All participants provided written informed consent to participate in the study before undergoing any study procedures. There were amendments to the protocol with regard to the objectives and statistical methods; these did not affect the study flow or outcome.

### 2.1 Participants

Eligible participants were aged 18–85 years and in good general health. They had complete removable dentures fitted to the upper and lower arches that were well fitting, as judged by the examiner using the Kapur Index (Olshan modification) for the evaluation of denture retention and stability (see below) (Olshan, Ross, Mankodi, & Melita, 1992; Kapur, 1967). Well‐fitting dentures were defined as having a Kapur Index sum score of ≥6 (upper and lower dentures combined (Olshan et al., 1992)). In addition, both upper and lower dentures had to be well made, defined as having adequate vertical dimension, freeway space, horizontal occlusal relationships and border extension, with acceptable porosity, tissue surfaces, polished surfaces, color and thickness. Eligible participants also reported experience of food trapping under their dentures during eating, which was demonstrated with a peanut particle migration rating of >0 for both dentures at screening (see below).

Participants were excluded from the study if they were pregnant or breastfeeding; were taking a medication that might interfere with study participation (including bisphosphonates); had a history of swallowing difficulties or choking; had any condition or were taking any medication that was currently causing, or was known to cause, xerostomia; had evidence of stomatitis, open sores, lesions, redness or swelling on oral soft tissue (OST) examination; or had an allergy or intolerance to the study materials/ingredients or to peanuts or any other nuts.

### 2.2 Procedures

The study comprised a screening visit and three test visits. At the screening visit, participants underwent an OST examination and were assessed for adequate food (peanut particle) migration under their dentures, as follows. Participants consumed a 30–32 g portion of peanuts then rinsed their mouth with water for 5 s before removing their dentures. The extent and location of peanut particle migration under each denture were visually assessed by an examiner using a 4‐point scale (0 = none, with no peanut migration under the denture; 1 = minimal, with slight migration under the denture; 2 = moderate, with migration of peanuts over the internal walls of the denture; 3 = extensive, with peanut migration on the crest of the denture). To ensure consistent examiner evaluation, photographs were taken of the underside of each denture immediately after denture removal in a subgroup of participants, selected at the discretion of the investigator, with the aim of obtaining photographs of at least two upper and two lower dentures within each assessment grade.

Participants with evidence of adequate peanut particle migration under either the upper or lower denture (score of >0 for either denture) were randomized (1:1:1) in a crossover fashion to receive one of the following treatments on each of three test days: 1) experimental denture adhesive application, referred to as the ‘test adhesive’, applied with a precision applicator as one continuous strip along the depth of the maxillary denture ridge (inside the buccolabial and posterior borders), with two smaller strips applied in the middle of the palatal area and one continuous strip along the depth of the mandibular denture ridge; 2) a standard marketed denture adhesive, referred to as the ‘positive control’ (Super Poligrip^®^ Free Denture Adhesive Cream; GSK Consumer Healthcare, Weybridge, UK [GSKCH]), applied using a flat ribbon nozzle as three dabs to the upper denture and two dabs to the lower denture as per the manufacturer's instructions; 3) no adhesive, referred to as the ‘negative control’. Both the test adhesive and positive control contained polyvinylmethyl ether/maleic acid, carboxymethylcellulose, petrolatum and mineral oil. Both denture adhesives had identical color, shape, and consistency.

As this was a crossover study, participants were assigned to the order in which they were to receive each treatment per a computer‐generated randomization schedule supplied by the Biostatistics Department of the study sponsor, using validated software (SAS Version 9.4; SAS Institute Inc., Cary, NC, USA). Randomization numbers were assigned in ascending numerical order as each participant was confirmed as fully eligible for study inclusion. The examiner performing the safety assessments and the laboratory staff who collected and weighed the peanut particles were blinded to the treatments.

**Figure 1:**
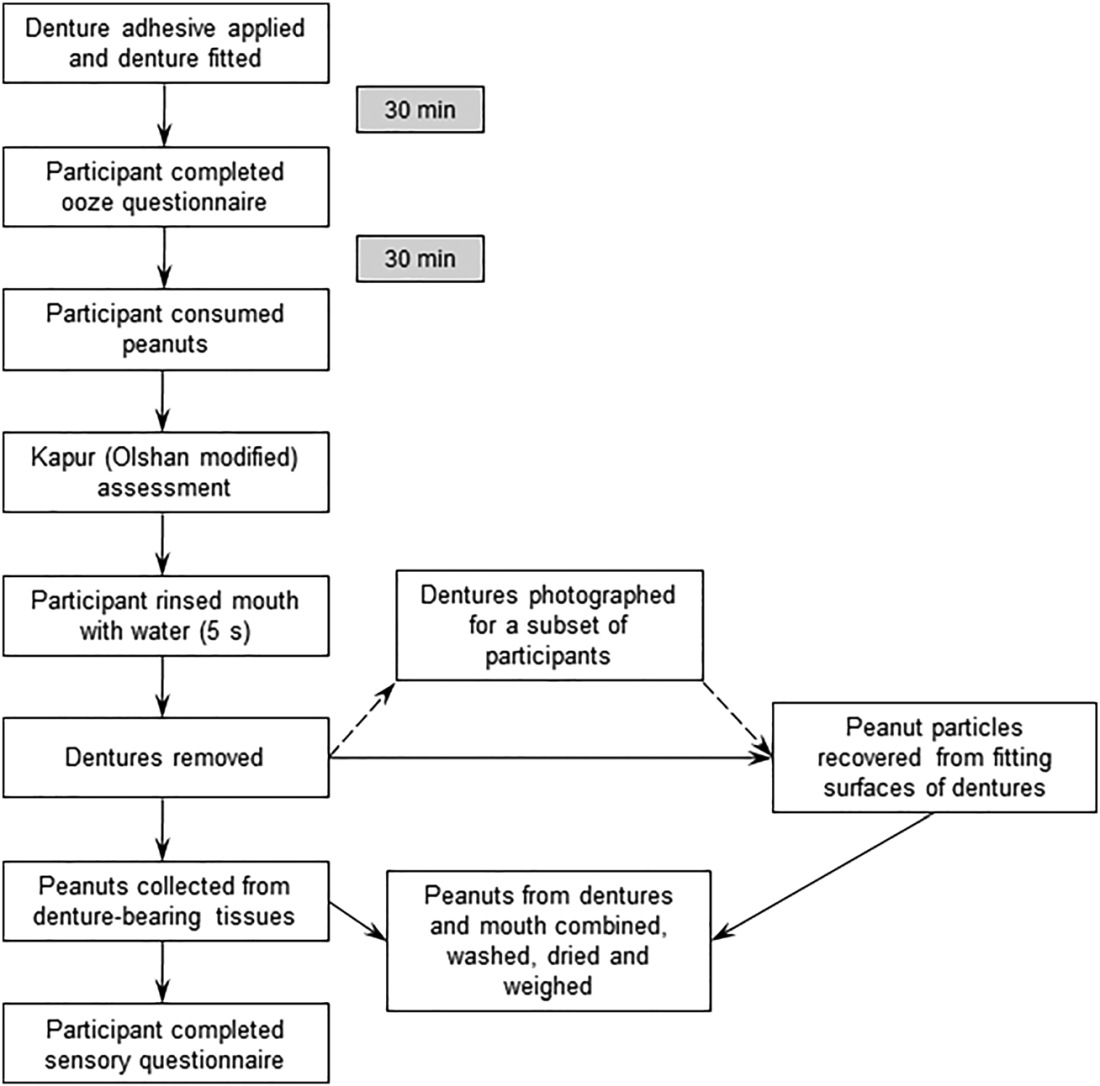
Flow diagram illustrating the clinical methodology for the efficacy measurements performed on treatment days

A flow diagram summarizing the clinical procedure is shown in Figure 1. On each test day, participants underwent an OST examination and their dentures were cleaned, dried and weighed. Treatment (or no treatment, as per the randomization schedule) was then applied to the upper and lower dentures by a member of staff at the study site in accordance with the study protocol. The dentures with applied adhesive were re‐weighed and the mass of the adhesive used was calculated as the difference in the recorded weights. The upper and lower dentures were returned to the participant, who positioned them in their mouth.

Approximately 30 min after inserting their dentures, participants who were randomized to either of the two denture‐adhesive groups for that visit completed a questionnaire on the oozing properties of the adhesive. At 60 (±5) min after inserting their dentures, all participants underwent food‐occlusion testing using the peanut particle migration method (see below). The retention and stability of the dentures, after chewing peanuts and before denture removal, were assessed by the examiner using the Kapur Index (Olshan modification). Immediately after denture removal, participants answered questions regarding their perceptions of denture stability and comfort, and their confidence and satisfaction with their dentures while chewing the peanuts. This was followed by a further OST examination. These procedures were repeated in a crossover fashion with at least 2 days between visits, to minimize any crossover effect between treatments, and a maximum of 7 days.

### 2.3 Efficacy measurements

#### 2.3.1 Peanut particle migration

Participants were instructed to chew a standardized portion of peanuts (30–32 g, accurately weighed), divided into approximately 4 g portions. Each portion was chewed for at least 20 s and then swallowed when comfortable to do so. Small sips of water were permitted during peanut consumption to aid chewing. After finishing the peanuts, participants rinsed their mouth with water for approximately 5 s to remove any peanut particles not retained under their dentures. The examiner then removed the lower denture and any peanut particles or adhesive remaining on the lower residual alveolar ridge were removed using gauze, which was retained. This process was repeated for the upper denture. Any residual peanut particles present on the surface of the dentures, other than those in contact with OST, were removed and discarded.

The upper and lower dentures plus corresponding gauze for each participant were placed in coded beakers (upper and lower separately) and approximately 100 mL of hot (90°C) deionized water, or sufficient to cover the dentures, was added to each beaker. The beakers were sonicated for 30 min to loosen any adhering peanut particles; any peanut particles still remaining on the dentures or the gauze were washed out into the beaker. The gauze pieces were discarded and the dentures were cleaned and returned to the participant. The solutions in the beakers (a mixture of water, adhesive, saliva and peanut particles) were heated to boiling point to dissolve any adhesive and then strained through a standard testing sieve. The remaining particles were washed repeatedly with hot water to remove any adhesive or saliva. After air‐drying overnight, the collected peanut particles were dried on pre‐weighed aluminium pans in an oven at 40°C for 5 h. The aluminium pans were then weighed to determine the mass of peanut particles collected from each denture.

**Figure 2:**
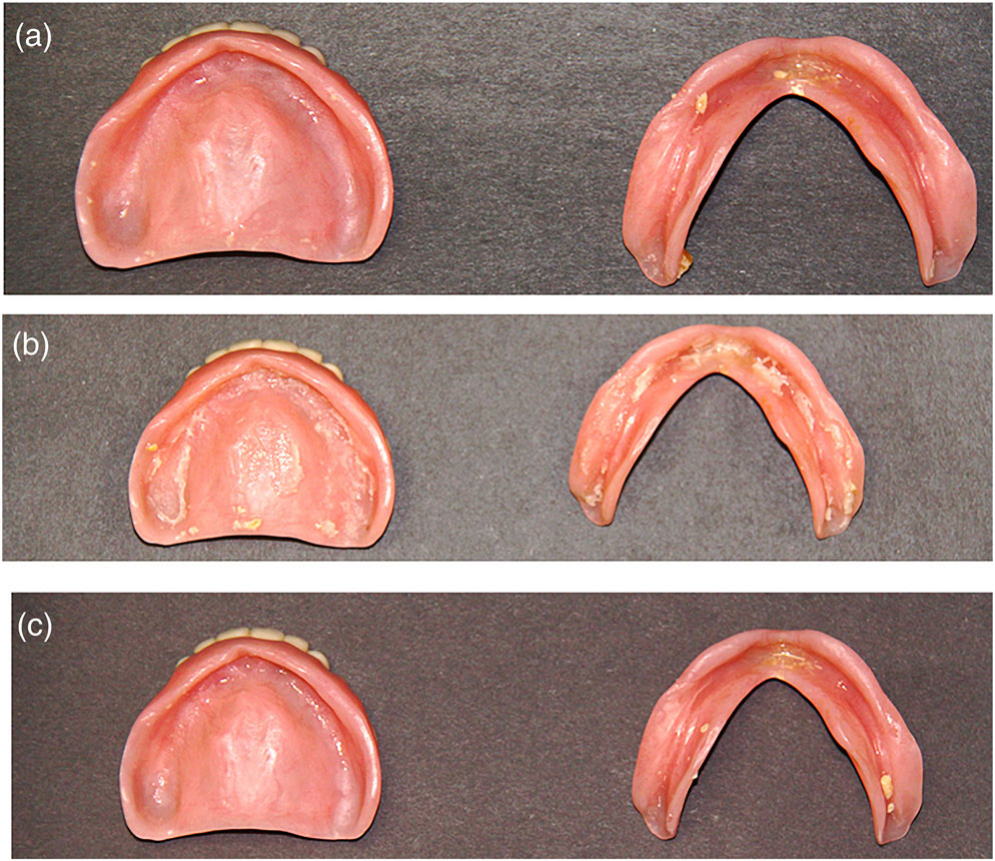
Photographs of dentures following the food‐occlusion testing showing peanut particles on the fitting surface from the same participant using (a) test adhesive, (b) negative control and (c) positive control

In a subset of participants (approximately five per treatment group), the denture fit surface was photographed before collection of the peanuts (Figure 2). These participants were selected at the discretion of the investigator, allowing for a variety of denture sizes and fits to be represented. These images were obtained for visualization purposes only and were not analyzed.

#### 2.3.2 Kapur Index for retention and stability

The Olshan modification of the Kapur Index (Coates, 2000; Kapur, 1967) was used to assess denture stability and retention at the screening visit and test visits. For the assessment of retention, the examiner was required to attempt to unseat the upper and lower denture by applying an opposing vertical force at the canine/lateral incisor region of the denture. Retention was then scored on a 6‐point scale: 0 = No retention; 1 = Poor retention; 2 = Fair retention; 3 = Good retention; 4 = Very good retention; 5 = Excellent retention. For the assessment of stability, the examiner attempted to rock the seated dentures by placing alternate horizontal force at the cuspid and contralateral molar regions of the upper and lower dentures. Stability was scored on a 5‐point scale: 0 = No stability; 1 = Poor stability; 2 = Fair stability; 3 = Good stability; 4 = Excellent stability. The sum score (upper + lower denture) was rated as follows: <6 = poor retention and stability; 6–9 = fair retention and stability; 10–14 = good retention and stability; >14 = very good retention and stability.

#### 2.3.3 Adhesive ooze

Approximately 30 min after inserting their dentures, and before undergoing food‐occlusion testing, participants randomized to the denture adhesive groups were asked to specify how long after denture insertion they had experienced denture adhesive oozing on a 5‐point scale (where 0 = immediately; 1 = <10 min; 2 = 10–20 min; 3 = 20–30 min; 4 = no ooze experienced).

#### 2.3.4 Confidence, comfort, satisfaction and denture movement

Immediately after denture removal following the food‐occlusion testing, participants answered four questions using 5‐point scales on their perceptions of: denture confidence (1 = not at all confident to 5 = extremely confident), comfort (1 = not at all comfortable to 5 = extremely comfortable), satisfaction (1 = not at all satisfied to 5 = extremely satisfied) and movement (1 = no movement at all to 5 = extremely high amount of movement). Participants rated their upper and lower dentures separately.

### 2.4 Safety assessments

OST examination findings at the screening visit and before and after the completion of each treatment assessment were used in the safety assessment. Any abnormalities reported from the start of the food migration assessment at screening until 5 days following the last administration of study product were reported as adverse events (AEs). Participant‐reported AEs and incidents were also reported.

### 2.5 Statistical analyses

It was planned to enroll approximately 100 participants to ensure 90 would be randomized and at least 82 evaluable participants would complete the entire study. It was estimated that this sample size would provide 90% power to detect a mean difference of 0.015 g between the test adhesive and the positive control in the mean weight of peanut particles recovered from full dentures, with a standard deviation (SD) for the paired differences of 0.04138 g and a correlation coefficient of 0.5. This sample size was estimated after consideration of data from a previous study (Munoz‐Viveros, Tyson‐Johnson, et al., 2011) using a paired *t* test and an α level of significance of 0.05. The smallest difference (test adhesive versus positive control) was considered for the sample size calculation. Analysis was carried out using SAS Version 9.4.

The primary efficacy analysis was based on the intent‐to‐treat population, which included all participants who were randomized, received at least one dose of study treatment and had at least one post‐baseline efficacy assessment. The safety population included all participants who were randomized and received at least one dose of study treatment.

The analysis of food occlusion (primary and secondary analyses) was performed on the combined masses of peanut particles from the upper and lower dentures using an analysis of variance (ANOVA) model with treatment and period as fixed effects and participant as a random effect. Two‐sided 95% confidence intervals (95% CI) were constructed and *P*‐values are reported. Adjusted means and standard errors (SE) for each treatment group were also calculated.

The Kapur Index (Olshan modification), denture adhesive ooze and subjective responses on denture comfort, confidence, satisfaction and movement were analyzed using ANOVA models as described for the primary analysis. Analyses were performed separately for upper and lower dentures.

OST abnormalities were included as AEs if they appeared or worsened after the initial assessment. No inferential analysis was performed to compare treatments with respect to safety.

## 3 RESULTS

**Figure 3:**
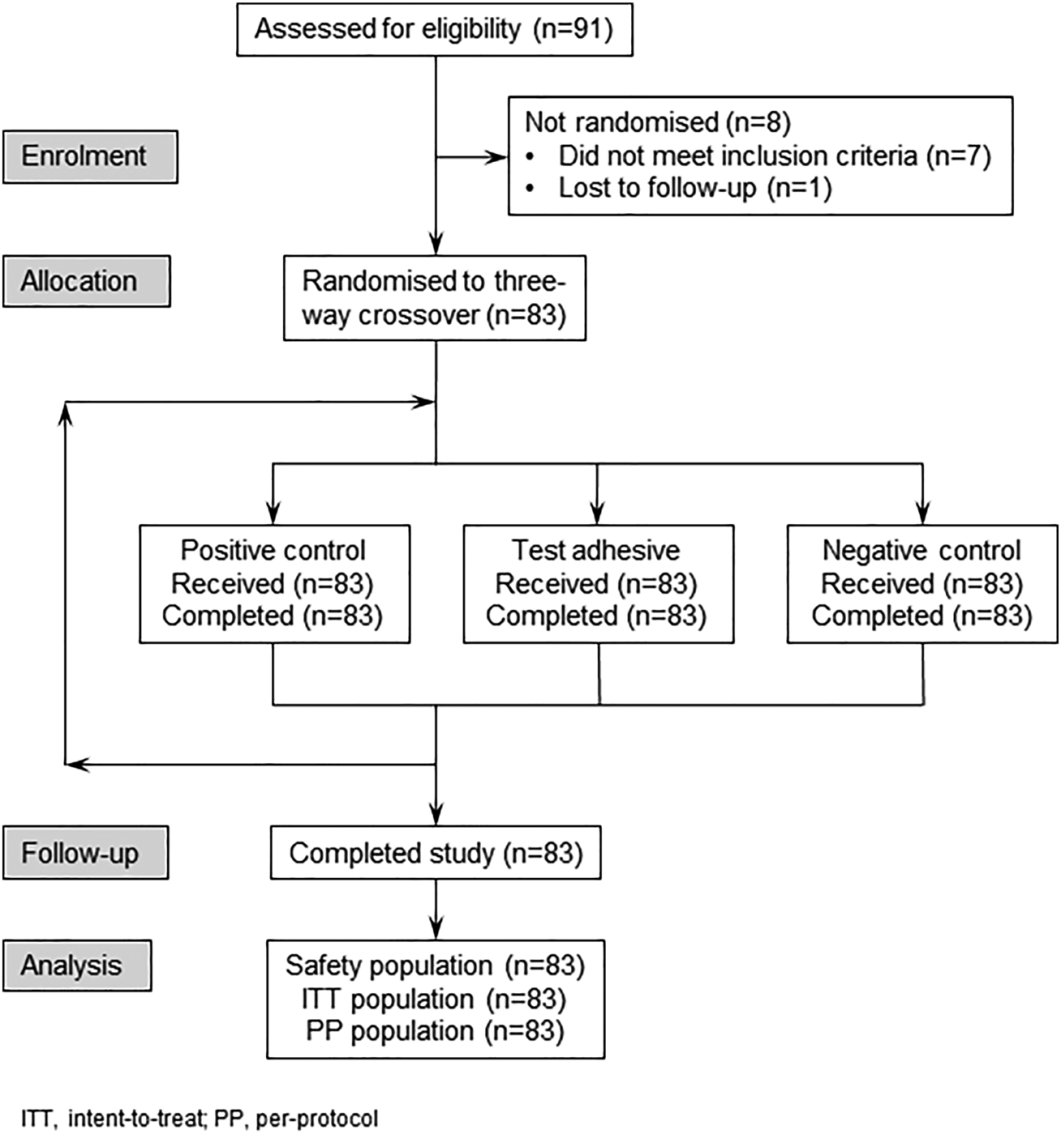
Participant disposition. ITT, intent to treat; PP, per protocol

A total of 91 participants were screened and 83 were randomized to and completed treatment (Figure 3). Most participants were women (*n* = 62, 74.7%) and were white (*n* = 70, 84.3%). The mean age was 63.4 years (SD 12.99; range 28–85 years). Denture history was similar for the upper and lower dentures, with mean denture ages of 9.5 and 9.3 years, respectively (Table 1). Overall, 52% of participants were completely satisfied with the fit of their upper denture, and 24% were completely satisfied with the fit of their lower denture.

**Table 1:** Denture history (all randomized population)

Characteristic	Upper denture	Lower denture
Length of time wearing a denture, years: mean (SD) [range]	19.9 (18.44) [0.2–67.0]	19.2 (18.18) [0.2–67.0]
Age of current dentures, years: mean (SD) [range]	9.5 (10.52) [0.2–50.0]	9.3 (10.57) [0.2–50.0]
Dentures relined, yes/no: n (%)	22 (26.5)/61 (73.5)	22 (26.5)/61 (73.5)
Dentures kept in mouth during sleep, yes/no: n (%)	38 (45.8)/45 (54.2)	29 (34.9)/54 (65.1)
Participant noticed recent changes in denture fit, yes/no: n (%)	13 (15.7)/70 (84.3)	19 (22.9)/64 (77.1)
Use denture adhesive to secure dentures, yes/no: n (%)	38 (45.8)/45 (54.2)	42 (50.6)/41 (49.4)
Satisfaction with denture fit: n (%)		
Completely satisfied	43 (51.8)	20 (24.1)
Somewhat satisfied	32 (38.6)	38 (45.8)
Somewhat dissatisfied	5 (6.0)	16 (19.3)
Completely dissatisfied	3 (3.6)	9 (10.8)
Food gets under the denture, yes/no: n (%)	83 (100)/0	83 (100)/0

SD, standard deviation

### 3.1 Peanut particle weight

The adjusted mean total weight (SE) of peanut particles recovered from the upper and lower dentures combined for the positive control, test adhesive and negative control groups is shown in Table 2. There were no statistically significant differences between any of the groups in terms of the mass of peanut particles recovered, including the primary objective of a difference between the positive control and negative control.

**Table 2:** Adjusted mean total weight of denture adhesive and peanut particles recovered from the upper and lower dentures combined (intent‐to‐treat population)

	Positive control	Test adhesive	Negative control
Denture adhesive, g: mean (SE) [range]	0.63 (0.018) [0.3–1.0]	1.05 (0.024) [0.6–1.5]	–
Recovered peanut particles, g: adjusted mean (SE)	0.06 (0.012)	0.07 (0.012)	0.08 (0.012)
	Comparison of recovered peanut particle weight (g)		
	Difference^a^	95% CI	*P*‐value
Positive control vs negative control	−0.02	−0.05, 0.00	.0987
Test adhesive vs negative control	−0.01	−0.04, 0.02	.6654
Test adhesive vs positive control	0.02	−0.01, 0.05	.2214

The analysis was performed using an analysis of variance model with mass of peanut particle (food occlusion) as response variable, treatment and period as fixed effect and participant as random effect.

a Difference is first named treatment minus second named treatment; negative differences favor the first named treatment.

CI, confidence interval; SE, standard error

### 3.2 Kapur Index (Olshan modification) retention and stability scores

Using the Kapur Index (Olshan modification), both the positive control and test adhesive scored ‘very good’–‘exellent’ (range 4.05–4.90) for upper‐ and lower‐denture retention and ‘good’–‘excellent’ (range 3.37–3.94) for upper‐ and lower‐denture stability (Table 3). The test adhesive had statistically significantly higher scores compared with the positive control for lower‐denture retention and stability both individually (both *P* < .001) and as a composite score (*P* < .0001, data not shown). The negative control scored ‘very good’–‘excellent’ for upper‐ and ‘good’–‘very good’ for lower‐denture retention, and ‘good’–‘excellent’ for upper‐ and ‘fair’–‘good’ for lower‐denture stability. Both denture adhesives were statistically significantly superior to the negative control in terms of retention and stability scores for both the upper and lower dentures (all *P* < .01) and for the composite scores (both *P* < .0001; data not shown).

**Table 3:** Kapur Index (Olshan modification) retention and stability scores (intent‐to‐treat population)

Parameter	Adjusted mean score (SE)			Difference (95% CI)		
	Positive control	Test adhesive	Negative control	Positive control vs negative control	Test adhesive vs negative control	Test adhesive vs positive control
Upper‐denture retention score	4.80 (0.060)	4.90 (0.060)	4.63 (0.060)	0.17 (0.05, 0.29) *P* = .0072	0.28 (0.15, 0.40) *P* < .0001	0.11 (−0.01, 0.23) *P* = .0834
Lower‐denture retention score	4.05 (0.106)	4.43 (0.106)	3.46 (0.106)	0.59 (0.40, 0.78) *P* < .0001	0.98 (0.79, 1.17) *P* < .0001	0.38 (0.19, 0.57) *P* < .0001
Upper‐denture stability score	3.88 (0.047)	3.94 (0.047)	3.72 (0.047)	0.16 (0.06, 0.25) *P* = .0015	0.22 (0.12, 0.31) *P* < .0001	0.06 (−0.04, 0.16) *P* = .2162
Lower‐denture stability score	3.37 (0.084)	3.67 (0.084)	2.90 (0.084)	0.47 (0.31, 0.63) *P* < .0001	0.77 (0.61, 0.93) *P* < .0001	0.30 (0.14, 0.46) *P* = .0002

The analysis was performed using an analysis of variance model with mass of peanut particle (food occlusion) as response variable, treatment and period as fixed effect and participant as random effect.

Difference is first named treatment minus second named treatment; positive differences favor the first named treatment.

CI, confidence interval; SE, standard error.

### 3.3 Participant perception and preference

**Figure 4:**
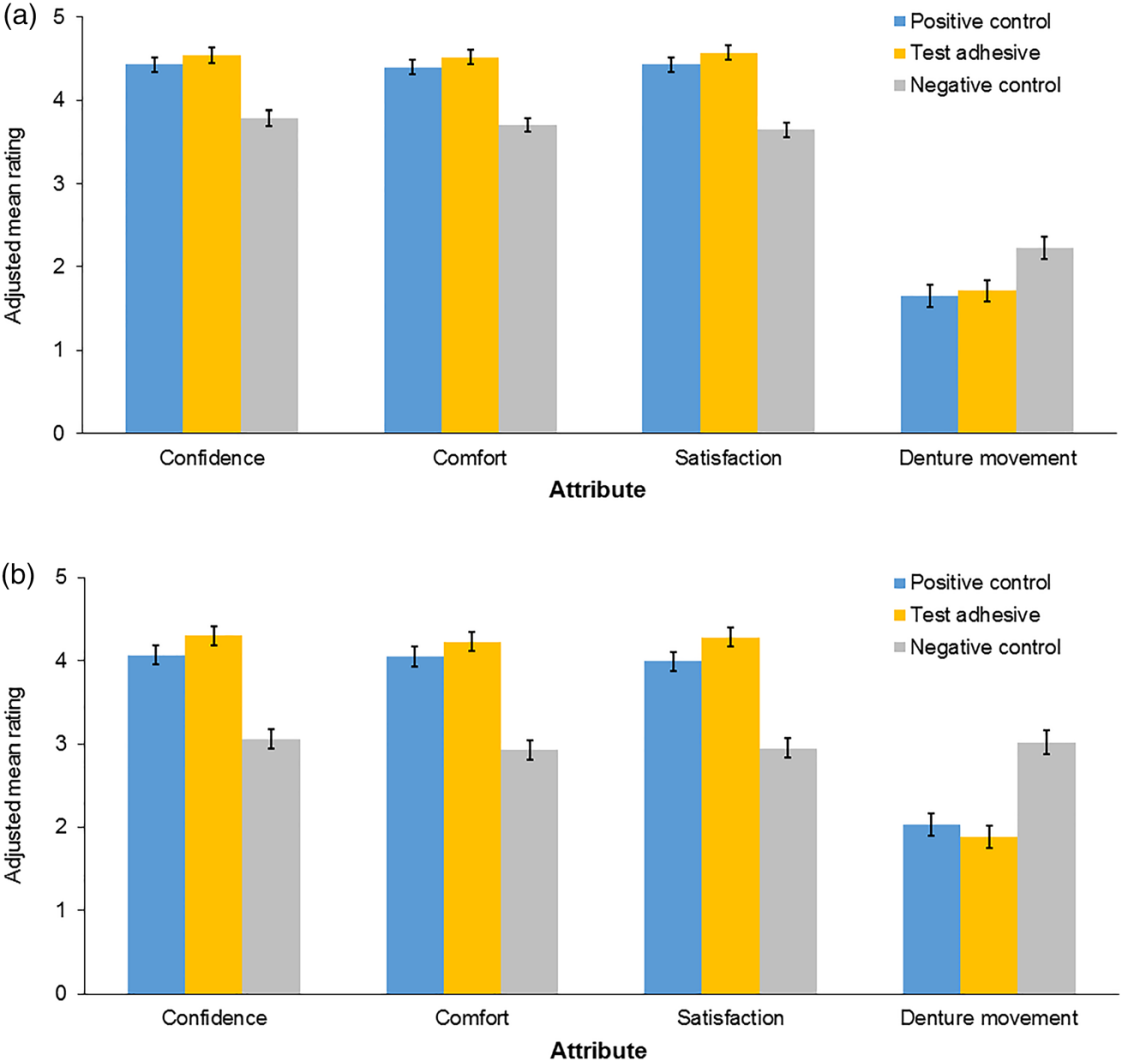
Adjusted mean (standard error) ratings in participants' evaluations of (a) upper dentures and (b) lower dentures (intent‐to‐treat population)

Adjusted mean scores are shown in Figure 4. Participants reported statistically significantly higher scores for confidence, comfort and satisfaction (mostly above 4, ‘very confident/comfortable/satisfied’) and statistically significantly lower scores for movement (mostly below 2, ‘slight movement’) with both the test adhesive and positive control compared with the negative control (scores of mostly around 3 for confidence/comfort/satisfaction; above 2 for movement) for both the upper and lower dentures (all *P* < .005) (Table 4). A statistically significant difference was observed in favor of the test adhesive versus the positive control for the criterion of ‘satisfaction’ for the lower dentures (*P* < .05).

**Table 4:** Difference between treatments for participant perception and preference for dentures (intent‐to‐treat population)

Parameter	Difference (95% CI)		
	Positive control vs negative control	Test adhesive vs negative control	Test adhesive vs positive control
Upper‐denture confidence	0.64 (0.41, 0.86) *P* < .0001	0.75 (0.52, 0.97) *P* < .0001	0.11 (−0.12, 0.34) *P* = .3409
Lower‐denture confidence	1.01 (0.75, 1.28) *P* < .0001	1.24 (0.98, 1.51) *P* < .0001	0.23 (−0.03, 0.49) *P* = .0884
Upper‐denture comfort	0.69 (0.50, 0.88) *P* < .0001	0.81 (0.62, 1.00) *P* < .0001	0.12 (−0.07, 0.31) *P* = .2099
Lower‐denture comfort	1.12 (0.86, 1.39) *P* < .0001	1.30 (1.04, 1.57) *P* < .0001	0.18 (−0.08, 0.45) *P* = .1775
Upper‐denture satisfaction	0.78 (0.56, 1.00) *P* < .0001	0.92 (0.70, 1.14) *P* < .0001	0.13 (−0.09, 0.36) *P* = .2311
Lower‐denture satisfaction	1.04 (0.78, 1.30) *P* < .0001	1.33 (1.07, 1.59) *P* < .0001	0.29 (0.03, 0.55) *P* = .0292
Upper‐denture movement	−0.58 (−0.09, −0.26) *P* = .0005	−0.52 (−0.84, −0.20) *P* = .0016	0.06 (−0.26, 0.38) *P* = .7200
Lower‐denture movement	−1.00 (−1.31, −0.69) *P* < .0001	−1.15 (−1.45, −0.84) *P* < .0001	−0.15 (−0.46, 0.16) *P* = .3451

The analysis was performed using an analysis of variance model with mass of peanut particles (food occlusion) as response variable, treatment and period as fixed effect and participant as random effect.

Difference is first named treatment minus second named treatment: positive differences favor the first named treatment for confidence, comfort and satisfaction; negative differences favor the first named treatment for denture movement.

CI, confidence interval.

### 3.4 Adhesive ooze

No statistically significant differences were observed between the test adhesive and the positive control for denture adhesive ooze for either the upper or lower dentures (Table 5).

**Table 5:** Denture adhesive ooze and comparison between treatments (intent‐to‐treat population)

Parameter	Adjusted mean score (SE)		Test adhesive vs positive control Difference (95% CI)
	Positive control	Test adhesive	
Upper‐denture adhesive ooze	3.82 (0.086)	3.75 (0.086)	−0.07 (−0.30, 0.16), *P* = .5395
Lower‐denture adhesive ooze	3.34 (0.154)	3.06 (0.154)	−0.27 (−0.65, 0.10), *P* = .1523

The analysis was performed using an analysis of variance model with mass of peanut particle (food occlusion) as response variable, treatment and period as fixed effect and participant as random effect.

Difference is first named treatment minus second named treatment; positive differences favor the first named treatment.

CI, confidence interval, SE, standard error

### 3.5 Safety

No treatment‐emergent AEs (TEAEs), treatment‐related TEAEs, serious AEs or medical device incidents were reported during the study.

## 4 DISCUSSION

This study did not find a statistically significant difference between the positive control (marketed adhesive) and the negative control (no adhesive) in peanut particle mass recovered from the upper and lower dentures combined. There was also no statistically significant difference in this measure between the test adhesive and the negative control or between the test adhesive and the positive control. Since the primary objective of the study was not met, the results cannot confidently be used to evaluate the effect of the denture adhesive application technique on food particles accumulation under the denture.

In a similar study where the amount of peanut particles retrieved was quantified, (Munoz et al., 2012) the authors concluded that the benefit associated with the use of adhesive could not be quantitatively differentiated from the no‐adhesive state. They attributed this to the very low weight of particles retrieved, which may have left very little room for improvement. However, the lack of statistically significant differences in this study contrasts with previous food‐occlusion studies conducted in full‐denture wearers (Ahmad et al., 2010; Clark, 2012; Munoz‐Viveros, Tyson‐Johnson, et al., 2011). In these studies, there was a statistically significant reduction in peanut particle migration under dentures when a marketed (Ahmad et al., 2010; Munoz‐Viveros, Tyson‐Johnson, et al., 2011) or experimental (Clark, 2012) adhesive was used compared with no adhesive.

The current methodology was largely based on a quantitative model for food‐occlusion methodology that has been validated in previous studies (Clark, 2012; Munoz et al., 2012; Munoz‐Viveros, Schober, et al., 2011; Munoz‐Viveros, Tyson‐Johnson, et al., 2011). However, a key difference was the denture adhesive application method. In previous studies, application was controlled by using a pre‐weighed sample of adhesive dispensed onto the dentures using a syringe (1.0 g to the upper dentures and 0.6 g to the lower dentures, total 1.6 g). The adhesives in the current study were applied directly from the adhesive packaging by the study staff, following the relevant user instructions. Hence, although the mass of adhesive used was determined, it was not controlled. This aspect of the study design was intended to replicate typical consumer usage and to allow the effect of the different application methods (dabs versus a continuous strip) to be factored into. The mean mass of the positive‐control adhesive used for the upper and lower dentures combined was 0.63 g (range 0.3–1.0 g; SD 0.16 g). This dose was considerably lower, with much greater variability, than that used in previous studies (standardized and controlled at 1.5 g), which might have contributed to the finding of no statistically significant difference in this study.

In general, the weight of peanut particles collected in this study was somewhat greater than reported in previous studies (Clark, 2012; Munoz‐Viveros, Tyson‐Johnson, et al., 2011). However, some aspects of the procedure associated with peanut collection, such as the duration and vigor of water rinsing, the intra‐oral peanut collection methodology and the application of adhesive (as discussed above), may not have been optimal and may therefore have resulted in sub‐optimal results. A further study is planned to better control these clinical aspects with the aim of improving the resolution of this model.

While objectives associated with the peanut‐mass endpoints were not met, statistically significant improvements in denture retention and stability were observed in the examiner‐led assessment (Kapur Index, Olshan modification) for the positive control and the test adhesive compared with the negative control. Similar outcomes were shown for the participants' assessment, with statistically significant increases in confidence, comfort and satisfaction and a decrease in denture movement when either of the two adhesives were used, compared with the negative control. These results are in agreement with other studies that have shown, using both similar and different test methods and adhesive formulations, that denture adhesive use can augment the retention, stability and comfort of well‐fitting conventional dentures (Munoz et al., 2012; Chew, Boone, Swartz, & Phillips, 1985; Tarbet et al., 1980). Of note, however, while the study participants were not informed of the groups they were in, they could not be blinded as to whether denture adhesive was applied so it cannot be fully ruled out that perception was based on the presence of the denture adhesive as opposed to its properties. The examiner was fully blinded to group participation.

In conclusion, the absence of a statistically significant benefit with the positive control and test adhesive compared with no adhesive in terms of food particle ingress under dentures compromised the ability to evaluate the effect of the adhesive application technique. The failure to observe a statistically significant difference may be attributed to the methodology used in this study and in particular, to the adhesive application procedure. The findings from this study illustrate the importance of controlling dosing in this methodology in order to reduce dosing variability that may compromise the ability to differentiate between test treatments. Further studies will be required to better control the food‐occlusion methodology used in such investigations. A single application of the positive‐control adhesive or test adhesive was generally well tolerated.

## AUTHOR CONTRIBUTIONS

All authors contributed to the design and reporting of the study, and/or were involved in its conduct. All authors had access to the final study report, made contributions to the development of the manuscript, had final responsibility for the decision to submit and approved the submitted version.

## DISCLOSURE STATEMENT

This study was funded by GSK Consumer Healthcare, of which Mounir Atassi and Gary Burnett are employees. Kimberly Milleman and Jeffery Milleman are employees of Salus Research, and Susmita Sanyal is an employee of Syneos Health; both of these companies have received funding from GSK Consumer Healthcare.
